# A Meaning‐Centered Intervention for Undergraduate Women With High Weight and Shape Concerns—Replication of a Randomized Controlled Trial

**DOI:** 10.1002/erv.3175

**Published:** 2025-01-31

**Authors:** Franziska Schutzeichel, Marije aan het Rot, Sanne F. W. van Doornik, Klaske A. Glashouwer, Mirjam I. Frey, Peter J. de Jong

**Affiliations:** ^1^ Department of Clinical Psychology and Experimental Psychopathology University of Groningen Groningen Netherlands; ^2^ Department of Eating Disorders Accare Child and Adolescent Psychiatry Groningen Netherlands

**Keywords:** comorbid, eating disorders, intervention, meaning, replication

## Abstract

**Objective:**

Recent studies underscore the relevance of life meaning to the maintenance of eating disorders. A previously conducted randomized controlled trial tested a meaning‐centered intervention for female university students with high weight and shape concerns. After a 6‐week online intervention led by a trainer, participants in the intervention condition scored higher on life meaning and lower on eating disorder symptoms and general distress compared to a waitlist group.

**Method:**

Given that the original study took place during COVID‐19, this study replicated the design to test the findings' robustness.

**Results:**

Compared to the waitlist condition (*n* = 68), participants in the intervention condition (*n* = 63) again scored higher on the presence of life meaning at post‐assessment and follow‐up. Participants in the intervention condition also showed moderately lower internalizing symptoms at both timepoints, whereas eating disorder symptoms were only reduced in those with relatively high baseline symptom severity.

**Conclusions:**

Thus, also without social distancing measures, the intervention increased life meaning and reduced eating disorder symptoms and comorbid internalizing symptoms in women with weight and shape concerns.

**Summary**

- After a meaning‐centered intervention participants reported more life meaning.
- Participants also reported lower EDE‐Q global scores.
- Post‐intervention they also reported lower levels of internalizing symptoms.

## 1 Introduction

Eating disorders pose a substantial burden on patients, including physical health problems, diminished quality of life, and cognitive and social impairments (van Doornik et al. 2021; van Hoeken and Hoek 2020). Currently, treatment outcomes remain suboptimal for many patients, with fewer than half of patients with anorexia nervosa (Treasure et al. 2015), bulimia nervosa, or binge eating disorder (Kessler et al. 2013) achieving long‐term recovery. In addition, comorbid mental disorders, particularly internalizing disorders like anxiety and depression (Udo and Grilo 2019), affecting 56%–95% of adults with eating disorders (Hudson et al. 2008; Keski‐Rahkonen and Mustelin 2016), further complicate treatment.

Previous research consistently links eating disorder symptoms with low life meaning (Fox and Leung 2009; Marco, Canabate, and Perez 2019; van Doornik et al. 2021, 2022). Life meaning can be viewed as a unidimensional concept, indicating the degree to which an individual perceives their life as meaningful (Steger et al. 2006), or from a tripartite perspective (George and Park 2016a; Martela and Steger 2016), distinguishing between comprehension (feeling that life makes sense), purpose (having goals in life), and mattering (the perception that one's life matters on a greater scale). The Meaning‐Making Model of Eating Disorders (MMMED; Marco et al. 2020) posits that cognitions and behaviors related to eating disorders can temporarily substitute for life meaning when individuals struggle to find meaning in normative domains like friendships and school. However, because efforts to regulate eating and weight often fail in the long term, dissatisfaction in these areas typically persists, leading to a lack of life meaning. This lack has been proposed as a factor contributing to the frequent comorbidity between eating disorders and internalizing disorders (Goodman, Doorley, and Kashdan 2018; Schutzeichel et al. 2024).

Conversely, experiencing life meaning can serve as a protective factor for mental health, contributing to increased well‐being and psychological functioning (Glaw et al. 2017). Several authors proposed that adding a component focused on increasing life meaning could benefit eating disorder treatments (Marco et al. 2020; Schutzeichel et al. 2024; van Doornik et al. 2021; de Vos et al. 2017). Therapeutic approaches that help increase life meaning have also been shown to be effective in decreasing psychological distress in patients with depression, advanced cancer, and other mental and physical disorders (Breitbart et al. 2018; Vos and Vitali 2018). These outcomes suggest that an added focus on life meaning might also reduce comorbidities in eating disorders.

Following this, van Doornik et al. (2024) developed a meaning‐centered intervention targeted at increasing life meaning and reducing eating disorder symptoms and general distress. In a randomized controlled trial (RCT), young women with weight and shape concerns received six weekly online intervention sessions. The sessions were led by a trainer and based on a workbook including homework exercises. The intervention aimed to increase life meaning by redirecting resources away from eating disordered cognitions and behaviors towards positive and sustainable goals and values. After the intervention, participants in the intervention condition scored higher in life meaning than the waitlist condition (*η*_*p*_^*2*^ = 0.25), also when separated into comprehension, purpose, and mattering, with moderate effects maintained after 4 weeks (*η*_*p*_^*2*^ = 0.11). Importantly, individuals in the intervention condition also reported lower levels of eating disorder symptomatology, as well as lowered general distress.

This study by van Doornik et al. (2024) provided first support for positive outcomes of meaning‐focused interventions on eating disorder symptomatology. However, replication is warranted to avoid premature large‐scale implementation, given the replication issues in previous psychological studies (Maxwell, Lau, and Howard 2015; Shrout and Rodgers 2018). Replicating van Doornik et al.'s (2024) findings is particularly relevant as their study took place during the acute phase of COVID‐19 when social distancing measures were in place. Research has shown that loneliness and social isolation increased during the pandemic (Buecker and Horstmann 2021; Dahlberg 2021); the large effect sizes of the intervention might therefore be an overestimation based on one‐on‐one human interaction during a time of social deprivation.

Therefore, we replicated the RCT to test the efficacy of the meaning‐centered intervention for increasing life meaning and reducing eating disorder and internalizing symptoms in young women with weight and shape concerns during a time without social distancing measures. Consistent with the original study, our primary aim was to examine whether the intervention led to increases in life meaning. We hypothesized that participants in the intervention condition would show higher scores in life meaning compared to participants in the waitlist condition at post‐assessment and follow‐up. As a secondary aim, we examined whether the effects of the intervention would hold for the separate concepts of meaning, that is, comprehension, purpose, and mattering. Additionally, we hypothesized that those in the intervention condition would also report lower scores in eating disorder symptoms and comorbid internalizing symptom measures than those in the waitlist condition at post‐assessment and follow‐up.

## 2 Method

This study was part of a larger project with two aims (AsPredicted #111500, https://aspredicted.org/BCC_F93). This article addresses the first aim of replicating an RCT by van Doornik et al. (2024), assessing the efficacy of a meaning‐centered intervention, including the critical measures that were assessed in the original study. Analyses regarding internalizing symptoms were added to be in line with the original study. The additional measures covered in the preregistration refer to the second aim of the overall project, which will be addressed in a different manuscript (Schutzeichel et al. 2025).

### 2.1 Participants

Between October 2022 and February 2024, 539 female first‐year psychology students at the University of Groningen were screened online with the Weight Concern Scale, which measures weight and shape concerns (WCS; Killen et al. 1994). All five items were adjusted to equal a maximum score of 20, leading to a total range of 0–100 (cf. Jacobi, Abascal, and Taylor 2004). In line with van Doornik et al. (2024), we invited participants to the study if they answered “always” or “often” to the WCS item “Do you ever feel fat?” (*n* = 62) or had a total score ≥ 47 on the WCS (*n* = 23) or both (*n* = 140), and excluded students who were currently undergoing eating disorder treatment (*n* = 2).

Of the 225 invited participants, 166 (73.78%, mean age = 19.57, SD = 2.09) completed the baseline measures in English (*n* = 70), Dutch (*n* = 82), or German (*n* = 14). Those who did not fill out the baseline measures in their native language indicated, for example, Romanian (*n* = 8), Slovak (*n* = 5), or Greek (*n* = 3) as their native language. Participants were then randomized either to the intervention condition (*n* = 84), which could be followed in English (*n* = 39), Dutch (*n* = 38), or German (*n* = 7), or to the waitlist control condition (*n* = 82). Sample descriptive variables per condition are available in Table 1.

**TABLE 1:** Sample descriptive score per condition at baseline/screening.

	**Condition**	
	Intervention (*n* = 84)	Waitlist control (*n* = 82)
Age at T1		
Mean (SD)	19.34 (1.70)	19.80 (2.41)
Median	19.00	19.00
Range	17.00–27.00	17.00–33.00
BMI at T1		
Mean (SD)	22.52 (2.74)	24.09 (5.29)
Median	22.41	22.99
Range	16.56–35.43	16.79–53.96
WCS at screening		
Mean (SD)	58.53 (14.11)	55.30 (14.48)
Median	55.00	52.50
Range	30.00–93.33	25.00–88.33

Abbreviations: BMI, Body Mass Index; WCS, Weight Concern Scale.

**FIGURE 1:**
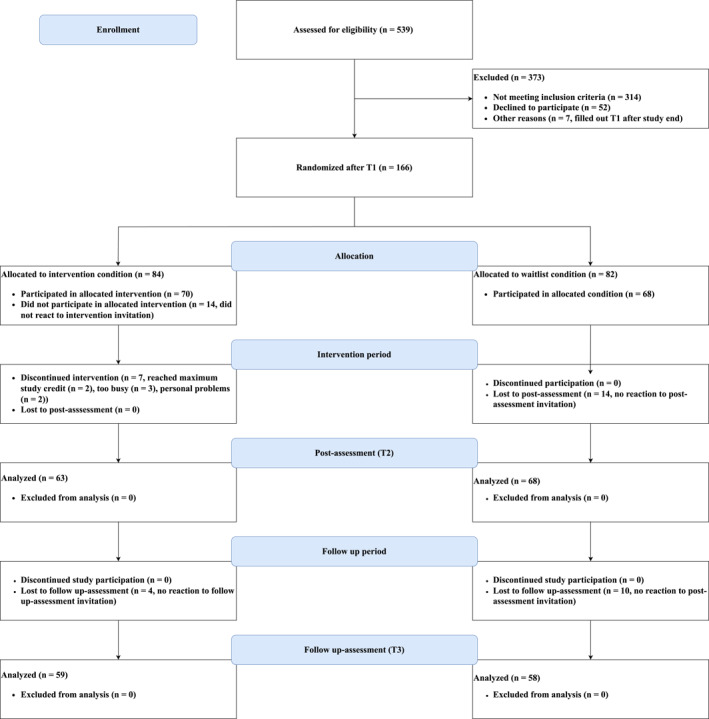
CONSORT flow diagram.

One hundred thirty‐one participants completed the post‐assessment (intervention *n* = 63, waitlist *n* = 68) and 117 participants completed the follow‐up assessment (intervention *n* = 59, waitlist *n* = 58). Figure 1 depicts the enrolment and participation flow of this study. Participants were reimbursed with partial course credits. Sample size was determined based on the a priori calculations used in van Doornik et al. (2024) with an *α*‐level of 0.05, a power of *β* = 0.80, an effect size of *f* = 0.25, number of groups = 2, number of covariates = 1, and numerator *df* = 1, leading to a required minimal sample size of *N* = 128.

### 2.2 Material

#### 2.2.1 Primary Measure

We used the Presence of Meaning in Life subscale of the Meaning in Life Questionnaire (MLQ; Steger et al. 2006) to assess life meaning from a unidimensional approach. This subscale has five items that are answered on a 7‐point Likert scale from 1 (absolutely untrue) to 7 (absolutely true). We calculated an average score of the subscale, in which a higher score indicated a higher presence of life meaning. Cronbach's *α* values varied between 0.89 and 0.90 for the different assessment timepoints, indicating good to excellent internal consistency.

#### 2.2.2 Secondary Measures

To measure life meaning from the tripartite view, we used the Multidimensional Existential Meaning Scale (MEMS; George and Park 2016b). The MEMS has 15 items, of which five each assess the three dimensions of Comprehension, Mattering, and Purpose on a 7‐point Likert scale from 1 (very strongly disagree) to 7 (very strongly agree). We averaged the items per subscale. High scores indicated high Comprehension, Purpose, or Mattering, respectively. Across timepoints, Cronbach's *α* values for the subscales varied between 0.83 and 0.92.

To evaluate the severity of core attitudinal eating disorder symptoms over the previous 4 weeks, we used the Eating Disorder Examination‐Questionnaire 6.0 (EDE‐Q; Fairburn and Beglin 2008). The EDE‐Q has 28 items of which 22 can be answered on a 7‐point Likert scale from 0 to 6, with varying anchors. The other six items are measured on an open scale and were not assessed. In line with Aardoom et al. (2012), we obtained a global score of the 22 items, with a higher score indicating more severe eating disorder psychopathology. Cronbach's *α* values varied between 0.91 and 0.95 across timepoints.

Symptoms of comorbid internalizing psychopathology, namely depression, anxiety, and stress, were assessed with the Depression Anxiety Stress Scales‐21 (DASS‐21; Lovibond and Lovibond 1995). The DASS‐21 has 21 items with three subscales of seven items each, that are rated on a 4‐point scale from 0 (did not apply to me at all) to 3 (applied to me very much). Scores per subscale were summed, with higher scores indicating more severe symptoms and eventually added for a total score. Across the timepoints, Cronbach's *α* values varied between 0.78 and 0.93 for the subscales and between 0.90 and 0.93 for the complete scale.

#### 2.2.3 Meaning‐Centered Intervention for Young Women With Weight and Shape Concerns

The meaning‐centered intervention for young women with weight and shape issues aimed to enhance a sense of life meaning to facilitate a reduction in weight and shape concerns. This structured 6‐week program incorporated theory, discussions, exercises, and homework assignments focused on themes of life meaning and eating disorder symptoms. Sessions were conducted individually and online, lasting for about 1 hour with participants using mailed workbooks. The six sessions were provided in English, Dutch, or German and led by trained psychologists or trained master's students.

Van Doornik and colleagues (2024) adapted existing manuals of meaning‐centered psychotherapies (Breitbart et al. 2018; van der Spek et al. 2017) by (i) reducing the number of sessions, and (ii) updating the content to suit the target demographic of young women with weight and shape concerns. For instance, the four sources of life meaning by Frankl (1959) were presented as a toolbox with various “tools” to enhance meaning. In the current intervention, the four sources were described as (1) your personal life story, (2) dealing with life's limitations, (3) creating your own life, and (4) meaningful experiences. Each session focused on one of the four sources. The first session introduced the concept and sources of meaning and their relationship with eating disorder symptoms. Participants defined meaning, documented recent meaningful experiences, and watched a video on life meaning and eating disorders. As a homework exercise, participants were asked to write down at least one meaningful experience daily. The second session focused on environmental influences on their life story. Assignments included writing about positive and negative life experiences and lessons, memories tied to emotions like joy, sadness, and regret, and identifying important people in their lives. Participants also created a word web answering “Who am I?”. Homework involved creating an overview of significant experiences, memories, people, relationships, and habits that shaped them. The third session explored personal influences on their life story. Afterwards, participants created timelines of their past and future and discussed them with significant others. The fourth session addressed current limitations and coping strategies by identifying tools they already use effectively (tops) and those they wish to use more often (tips) and listing five short‐term goals. Homework involved choosing a goal, creating a step‐by‐step plan to achieve it, and taking the first step. In the fifth session, participants reflected on experiences of courage, responsibility, and commitment, and they discussed meaningful experiences through drawings or pictures. Homework included summarizing lessons learned in the intervention and advancing towards their chosen goal. In the final session, participants shared their life lessons and reflected on their intervention experience by answering guided questions​.

### 2.3 Procedure

The study was approved by the Ethics Committee of the Faculty of Behavioral and Social Sciences at the University of Groningen (PSY‐2223‐S‐0010) and was registered with ClinicalTrials.gov (NCT06462300).

After students gave online consent to the screening, they filled out the WCS, and those meeting the inclusion criteria were invited to participate in the study. Participants filled out a separate online informed consent form for the main study and then indicated their demographic information, followed by completing baseline (T1) measures in the same order as mentioned in the material section. Participants also answered additional questionnaires during each assessment, which were included for the second aim of the overall project (AsPredicted #111500) and which will be addressed in a separate article. After completion of T1, participants were randomly allocated to either the intervention or the waitlist condition (parallel design) through a random integer generator (Haahr 2024). The main researcher (FS) assigned the participants in the intervention condition to one of the trainers.

Immediately after the intervention, the participants were invited to fill out the post‐assessment (T2), which included the same questionnaires and order as T1. The waitlist control participants were invited to fill in T2 7 weeks after T1. Four weeks later, both groups received an invitation for the follow‐up assessment (T3, again with the same questionnaires and in the same order). The invitations for all questionnaires were sent out via e‐mail and participants received up to three reminders to complete the assessment. After completion of the data collection, participants in the waitlist condition were given the opportunity to follow the meaning‐centered intervention outside of the study. Hence, the only procedural difference with van Doornik et al. (2024) was that the participants were not affected by COVID pandemic‐related public health measures.

### 2.4 Analysis

We followed the same data‐analytical procedures as van Doornik and colleagues (2024). Accordingly, for our primary outcome measures, we preregistered two separate ANCOVAs to test the efficacy of the intervention for the presence of life meaning with Condition (intervention, waitlist) as a between‐subjects factor and the baseline MLQ‐P score as covariate, once with the post‐assessment score as dependent variable (*n* = 131) and once with the follow‐up assessment score as dependent variable (*n* = 117). These analyses were repeated with the secondary outcome measures including the MEMS subscales scores of Comprehension, Purpose, and Mattering, as well as the EDE‐Q global score and the DASS‐21 total score and subscales Depression, Anxiety, and Stress as outcome variables and the according baseline scores as covariate. To correct for familywise error rate, we applied Bonferroni‐Holm corrections to the secondary analyses, resulting in the smallest *p*‐value being tested against an *α* of 0.00625, followed by 0.00714, 0.00833, 0.01, 0.0125, 0.01667, 0.025, and 0.05.

To obtain additional insight into the effectiveness of the intervention (Silverman, Pettit, and Jaccard 2024), we complemented the main analyses with an intention‐to‐treat (ITT) approach by performing Predictive Mean Matching (PMM) in SPSS with five donors, 30 imputations, and up to 10 iterations (van Buuren 2018), including all participants who were randomized (*N* = 166). Meaningful differences in significance or effect size magnitude (e.g., from moderate to small) between completers and ITT analyses are reported. For all outcomes of these analyses and additional outcomes of a mixed linear modeling approach refer to the supplementary materials.

## 3 Results

Table 2 shows an overview of all variables per group and assessment timepoint, including mean, standard deviation, and median. All analyses regarding the primary and secondary aims showed large covariate effects, for exact statistical outcomes regarding the covariates refer to the supplementary materials.

**TABLE 2:** Overview of statistic properties of the materials per group and assessment timepoint.

	Intervention condition			Waitlist control condition		
	T1 (*n* = 84) Mean (SD) Median	T2 (*n* = 63) Mean (SD) Median	T3 (*n* = 59) Mean (SD) Median	T1 (*n* = 82) Mean (SD) Median	T2 (*n* = 68) Mean (SD) Median	T3 (*n* = 58) Mean (SD) Median
MLQ‐P	4.17 (1.30) 4.40	5.17 (1.05) 5.40	4.92 (1.17) 5.20	4.00 (1.14) 4.10	4.11 (1.18) 4.20	4.12 (1.09) 4.20
MEMS						
Comprehension	4.05 (1.06) 4.00	4.78 (0.96) 5.00	4.56 (0.91) 4.60	3.80 (0.87) 3.80	4.13 (0.93) 4.20	4.07 (0.95) 4.00
Purpose	4.97 (1.02) 5.00	5.53 (0.81) 5.60	5.40 (0.89) 5.60	4.80 (0.99) 5.00	4.86 (0.98) 5.00	4.91 (0.90) 5.00
Mattering	3.24 (1.22) 3.20	3.82 (1.14) 3.80	3.59 (1.15) 3.40	2.98 (1.06) 3.00	3.05 (1.18) 3.10	3.15 (1.28) 3.10
EDE‐Q^a^	2.98 (0.97) 2.91	2.00 (1.09) 1.91	2.03 (1.08) 2.23	2.95 (1.13) 2.89	2.87 (1.28) 2.89	2.85 (1.12) 2.75
DASS‐21						
Total	25.82 (12.27) 25.00	17.41 (11.15) 14.00	18.71 (11.58) 17.00	24.43 (10.84) 24.00	24.53 (10.29) 24.00	24.62 (11.76) 25.50
Depression	7.30 (5.17) 6.00	4.94 (4.34) 4.00	5.63 (4.56) 4.00	7.52 (5.01) 7.00	7.75 (4.86) 7.00	8.34 (5.23) 8.00
Anxiety	8.06 (4.84) 8.00	5.54 (4.38) 4.00	5.42 (3.79) 5.00	7.41 (4.46) 6.00	7.66 (4.20) 7.00	7.02 (4.12) 7.00
Stress	10.46 (4.11) 11.00	6.94 (4.33) 6.00	7.66 (4.45) 8.00	9.49 (4.38) 9.00	9.12 (4.25) 9.00	9.26 (4.83) 9.00

*Note:* This table serves as an overview of the available data per assessment.

Abbreviations: DASS‐21, Depression Anxiety Stress Scales‐21 (total score and subscales Depression, Anxiety, Stress); EDE‐Q, Eating Disorder Examination‐Questionnaire; MEMS, Multidimensional Existential Meaning Scale, subscales Comprehension, Purpose, Mattering; MLQ‐P, Meaning in Life Questionnaire Presence subscale.

^a^ In a psychometric study, a score of 2.29 reflected the 10th percentile of a Dutch population of treatment seeking individuals with an eating disorder (*N* = 935) and the 90th percentile of the general population (*N* = 235; Aardoom et al. 2012). 68.3% of our sample scored above this cut‐off at T1.

### 3.1 Assumption Testing

No assumption violations requiring a change in the preregistered ANCOVAs were detected. In case of outliers according to the Tukey fence method (Tukey 1977), the analyses were repeated without the outliers. Unless otherwise reported, removing outliers did not lead to relevant changes in significance testing. Information about differences between dropouts and completers and differences between languages can be found in the supplementary materials.

### 3.2 Primary Outcome: Presence of Life Meaning

The ANCOVA including post‐assessment MLQ‐presence as outcome variable demonstrated a large effect of Condition, *F*(1, 128) = 37.37, *p* < 0.001, *η*_*p*_^*2*^ = 0.23.

The analysis including follow‐up assessment MLQ‐presence as outcome variable showed a moderate effect of Condition, *F*(1, 114) = 12.04, *p* < 0.001, *η*_*p*_^*2*^ = 0.10. Following the ITT approach, the effect sizes decreased for post‐assessment (*F*(1, 163) = 20.35, *p* < 0.001, *η*_*p*_^*2*^ = 0.11) and follow‐up (*F*(1, 163) = 6.32, *p* < 0.01, *η*_*p*_^*2*^ = 0.04). Thus, in comparison to participants in the waitlist condition, those in the intervention condition reported significantly higher scores on the MLQ‐P immediately after the intervention and at follow‐up.

### 3.3 Secondary Outcomes

#### 3.3.1 Multidimensional Existential Meaning Scale

Condition demonstrated a moderate effect on the MEMS T2 scores: MEMS‐comprehension *F*(1, 128) = 15.15, *p* < 0.001, *η*_*p*_^*2*^ = 0.11; MEMS‐purpose *F*(1, 128) = 15.31, *p* < 0.001, *η*_*p*_^*2*^ = 0.11, and MEMS‐mattering *F*(1, 128) = 18.65, *p* < 0.001, *η*_*p*_^*2*^ = 0.13.

For none of the MEMS subscales the difference between conditions at T3 retained significance (MEMS‐comprehension *p* > 0.01, MEMS‐purpose *p* > 0.0125, MEMS‐mattering *p* > 0.025). However, removing six outliers for the follow‐up analysis on the MEMS‐purpose led to a significant outcome (*F*(1,104) = 60.55, *p* = 0.002 (compared against *α* = 0.007), *η*_*p*_^*2*^ = 0.09). In short, when compared to participants in the waitlist condition, participants in the intervention condition on average showed significantly higher scores in Comprehension, Purpose, and Mattering immediately after the intervention, but not reliably at follow‐up.

#### 3.3.2 Eating Disorder Symptoms

The assumption of homogeneous regression slopes was violated for the EDE‐Q analyses. To relax the assumption, we added the interaction effect EDE‐Q(T1)*Condition to the model (Cohen et al. 2003), resulting in a significant interaction with a moderate effect size, *F*(1, 127) = 9.43, *p* < 0.01, *η*_*p*_^*2*^ = 0.07, and a non‐significant effect of Condition on EDE‐Q scores at post‐assessment (*p* > 0.05). Comparison of slopes by means of regression analyses showed a significant difference between the conditions (*p* < 0.005), with *b* = 0.95 for T1 EDE‐Q for the waitlist condition and *b* = 0.49 for T1 EDE‐Q for the intervention condition.

**FIGURE 2:**
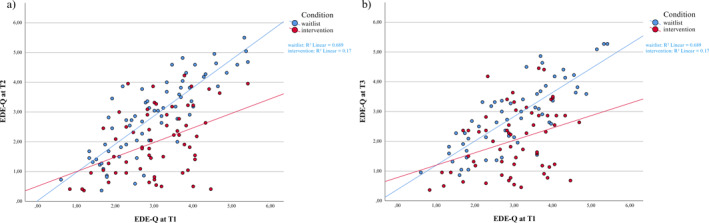
Scatterplots of Condition and T1 EDE‐Q Score per Timepoint. (a) EDE‐Q scores at T1 versus EDE‐Q scores at T2 per condition. (b) EDE‐Q scores at T1 versus EDE‐Q scores at T3 per condition. The flatter the slope, the more improvement from baseline to post‐assessment/follow‐up with increasing baseline scores.

Similar outcomes were found for the analysis including the follow‐up EDE‐Q score, with a non‐significant effect of Condition (*p* > 0.05), as well as a small EDE‐Q(T1)*Condition interaction effect, *F*(1, 113) = 5.80, *p* < 0.05, *η*_*p*_^*2*^ = 0.05. There was a significant difference in slopes between the conditions (*p* < 0.05), with *b* = 0.81 for the waitlist condition and *b* = 0.42 for the intervention condition. When following the ITT approach, there was a moderate effect of Condition at T2 (*F*(1,163) = 17.20, *p* < 0.001, *η*_*p*_^*2*^ = 0.10) and a small effect at T3 (*F*(1,163) = 10.20, *p* < 0.001, *η*_*p*_^*2*^ = 0.06). These results together with visual inspections of EDE‐Q at T1 in relation to EDE‐Q at T2/T3 (Figure 2) suggest that participants in the intervention condition who scored relatively high on the EDE‐Q at T1 had lower scores at T2 and T3 in comparison to those scoring lower at T1.

#### 3.3.3 Internalizing Symptoms

All analyses including the DASS total and DASS subscales with their respective T2 scores as outcome demonstrated moderate to strong effects of Condition: DASS total *F*(1, 128) = 24.27, *p* < 0.001, *η*_*p*_^*2*^ = 0.16, Depression *F*(1,128) = 13.29, *p* < 0.001, *η*_*p*_^*2*^ = 0.09, Anxiety *F*(1,128) = 16.40, *p* < 0.001, *η*_*p*_^*2*^ = 0.11, and Stress *F*(1,128) = 13.50, *p* < 0.001, *η*_*p*_^*2*^ = 0.10.

The analyses including the DASS total and DASS subscales with their respective T3 scores as outcome demonstrated moderate effects of Condition for the DASS total *F*(1, 113) = 8.96, *p* < 0.01, *η*_*p*_^*2*^ = 0.07, Depression *F*(1,114) = 7.51, *p* = 0.007, *η*_*p*_^*2*^ = 0.06, and Anxiety *F*(1,114) = 7.20, *p* = 0.008, *η*_*p*_^*2*^ = 0.06. Stress scores at follow‐up were not significantly impacted by Condition (compared against *α* = 0.01667), unless following the ITT approach (*F*(1,163) = 5.96, *p* = 0.01, *η*_*p*_^*2*^ = 0.04). The effect on the DASS total was slightly smaller (*F*(1,163) = 10.38, *p* = < 0.001, *η*_*p*_^*2*^ = 0.06) when using the ITT approach. These outcomes indicate that when compared to participants in the waitlist condition, participants in the intervention condition on average showed significantly lower scores in combined DASS total scores and when relying on the ITT principle, this also yields for all subscales at post‐assessment and follow‐up.

#### 3.3.4 Post‐Hoc Analyses

To assess the relation between changes in primary and secondary measures, we explored their bivariate correlations at post‐assessment and follow‐up (Table 3). As a high score in meaning‐related variables is desirable, while the opposite is true for symptom variables, changes in meaning‐related variables were calculated as T2/T3—T1 and changes in psychopathology symptoms as T1—T2/T3. Changes in life meaning‐related variables were mostly moderately to strongly correlated with changes in eating disorder and internalizing psychopathology at both timepoints. This demonstrates that the increase in life meaning through the intervention was overall significantly related to decreases in ED symptoms and internalizing psychopathology.

**TABLE 3:** Exploratory correlations between changes of primary and secondary outcome variables per timepoint.

Change in variable between T1 and T2	1.	2.	3.	4.	5.	6.	7.	8.
1. MLQ‐presence								
2. MEMS‐comprehension	0.54							
3. MEMS‐purpose	0.43	0.36						
4. MEMS‐mattering	0.47	0.50	0.31					
5. EDE‐Q	0.28	0.39	0.21	0.37				
6. DASS total	0.33	0.31	0.12	0.41	0.40			
7. DASS‐depression	0.32	0.34	0.27	0.34	0.27	0.72		
8. DASS‐anxiety	0.12	0.16	0.03	0.30	0.31	0.78	0.32	
9. DASS‐stress	0.32	0.22	0.00	0.33	0.35	0.84	0.36	0.54

Change in variable between T1 and T3	10.	11.	12.	13.	14.	15.	16.	17.
10. MLQ‐presence								
11. MEMS‐comprehension	0.62							
12. MEMS‐purpose	0.45	0.40						
13. MEMS‐mattering	0.53	0.49	0.45					
14. EDE‐Q	0.32	0.33	0.21	0.31				
15. DASS total	0.43	0.33	0.27	0.35	0.46			
16. DASS‐depression	0.45	0.38	0.33	0.34	0.44	0.81		
17. DASS‐anxiety	0.24	0.23	0.14	0.26	0.38	0.81	0.45	
18. DASS‐stress	0.37	0.22	0.19	0.29	0.33	0.88	0.56	0.62

Abbreviations: DASS‐21, Depression Anxiety Stress Scales‐21 (total score and subscales Depression, Anxiety, Stress); EDE‐Q, Eating Disorder Examination‐Questionnaire; MEMS, Multidimensional Existential Meaning Scale, subscales Comprehension, Purpose, Mattering; MLQ‐Presence, Meaning in Life Questionnaire Presence subscale.

We also explanatorily examined the intervention effects on the Eating Disorder Inventory‐2 Bulimia subscale (EDI‐B; Garner 1991; assessed for a different aim) using the same approach as for the preregistered analyses. At T2, condition had a moderate effect on the EDI‐B, *F*(1, 117) = 8.44, *p* < 0.01, *ηp* ^2^ = 0.07, but ITT analyses showed no significant effect (*p* > 0.05). At T3, condition had a large effect, *F*(1, 113) = 17.44, *p* < 0.001, *ηp* ^*2*^ = 0.13, but ITT analyses yielded a smaller effect size, *F*(1, 163) = 8.57, *p* < 0.01, *ηp* ^*2*^ = 0.05.

## 4 Discussion

The current study replicated a RCT by van Doornik et al. (2024) to investigate the efficacy of a meaning‐centered intervention for young women with weight and shape concerns under circumstances without social distancing measures. We examined the impact of the intervention on life meaning, eating disorder symptoms, and comorbid internalizing symptoms. In short, the main findings can be summarized as follows: Participants in the intervention condition showed significantly higher scores in life meaning than those in the control condition at both post‐assessment and follow‐up. Moderate effects at post‐assessment could be observed when dividing meaning into the separate concepts of comprehension, purpose, and mattering. Yet, the latter effects were not maintained at follow‐up. Participants in the intervention condition showed moderately lower internalizing symptoms at both timepoints, whereas the reduction in eating disorder symptoms was dependent on relatively higher baseline severity.

Our findings regarding meaning‐related outcome variables are in line with previous implementations of meaning‐centered interventions for several physical and psychological disorders showing improvements in life meaning (Breitbart et al. 2015; van der Spek et al. 2017; van Doornik et al. 2024), but also in quality of life in general and well‐being (Vos and Vitali 2018). The intervention had been designed to increase life meaning by developing a “toolbox” of meaning. This toolbox is used to derive meaning from (1) your personal life story, (2) dealing with life's limitations, (3) creating your own life, (4) and meaningful experiences. When investigating the efficacy of the intervention on separate meaning dimensions of comprehension, purpose, and mattering, it proved to be effective in the short‐term for all three dimensions. Thus, participants in the intervention condition felt as if their lives made more sense (comprehension), had goals to strive towards (purpose), and as if their lives mattered on a greater scale (mattering) immediately after the intervention. Reliable long‐term effects within the meaning measures were however only observed in presence of life meaning, a more general, unidimensional measure (Steger et al. 2006).

While the exact mechanisms underlying the increase in the tripartite dimensions remain to be investigated, it is conceivable that trying to create a coherent and personal life story based on life events and understanding how limitations influence one's own story can foster a greater sense of comprehension (McLean 2008). Understanding one's own life story potentially increases the understanding of the individual's environment, making the environment more predictable and creating a coherent picture of life (Martela and Steger 2016). Purpose might have been increased by targeting clear goals in line with participants' values to create their own life beyond a focus on weight and shape. Parts of the later sessions were specifically designed to create a step‐by‐step list to make both the purpose and higher‐order goals more tangible (George and Park 2016a). Lastly, the reflection on meaningful experiences might have enabled participants to perceive the significance of their life within the broader context of existence, by evaluating their experiences and the impact those have had on themselves and others (George and Park 2016a). Examining meaningful life events might have added to the extent to which participants felt significance and value in their existence, giving them a sense of “living well, successfully, and responsibly” (Martela and Steger 2016, 535).

Participants relatively high on a global measure of eating disorder symptoms at baseline appeared to derive more benefit from the intervention in terms of symptom reduction. Consistent with the findings by van Doornik and colleagues (2024), decreases in eating disorder symptoms at post‐assessment and follow‐up were strongly correlated with increases in life meaning. It is plausible that the focus on more positive and meaningful goals may have diverted attention away from weight and shape‐related goals (Williamson et al. 2004) or potentially decreased the motivational incentive of these goals (Cox, Klinger, and Fadardi 2015, cf. van Doornik et al. 2024). These possible mechanisms align with previous literature describing increased life meaning as a protective factor against psychopathological symptoms (Glaw et al. 2017) as well as the idea of the MMMED (Marco et al. 2021). Additionally, given the strong correlation between reductions in global eating disorder symptoms and decreases in internalizing symptoms, improvements in internalizing symptomatology may have also contributed to the observed changes as suggested by previous research (Mitchell et al. 2014). In conclusion, our findings further support the idea that integrating a meaning‐centered approach into existing treatment options for mental disorders could improve their outcomes (de Vos et al. 2017; van Doornik et al. 2024). Moreover, it might be worthwhile to consider this intervention in terms of its potential preventative effects on eating disorders, as intervening early in individuals with subclinical symptoms is one of the strongest predictors of improved treatment outcomes (Wade, Shafran, and Cooper 2023).

While specifically tailored to address eating disorder symptoms, the meaning‐centered intervention yielded broader benefits, resulting in lower scores of comorbid symptoms of depression, anxiety, and stress for the participants in the intervention condition. Previous RCTs already demonstrated the effectiveness of meaning‐centered interventions in alleviating anxiety and depressive symptoms across different populations (e.g., patients with advanced cancer, uncomplicated grief, women with fertility problems; Breitbart et al. 2015; MacKinnon et al. 2015; Mosalanejad and Khodabakshi Koolee 2013) despite not directly targeting these symptoms. Marco et al. (2021) suggested that life meaning may serve as a potential mediator between emotion dysregulation and psychopathology symptoms. Consequently, our intervention might have facilitated decreased internalizing symptoms by enhancing participants' sense of life meaning, thereby weakening the impact of emotion dysregulation on mental health. Engagement with one's own interpretation of life meaning potentially enabled participants who may have previously felt indifferent towards meaning or experienced a crisis of meaning (Chmielewski et al. 2022) to discover significance in various aspects of their lives. During the meaning‐centered intervention, many participants described goals that revolved around fostering deeper connections with friends and family or spending more time on enjoyable leisure activities. Previous research suggests that positive life events partially mediate the association between life meaning and depressive symptoms (Disabato et al. 2017). Hence, actively pursuing meaningful goals appears to lessen depressive symptoms. Furthermore, the pursuit of meaningful goals, rather than the attainment, is argued to serve as a mechanism through which individuals, including those with (social) anxiety, experience increased self‐esteem and greater psychological well‐being (Goodman, Doorley, and Kashdan 2018). This effect can also be seen in applications of Acceptance and Commitment Therapy (ACT), in which an emphasis lies on engagement with the present moment and striving for one's own values, even when psychological distress is present (Hayes and Pierson 1999).

Our study benefitted from an RCT design with a large sample size of young women with weight and shape concerns to investigate the stability of previous findings by van Doornik et al. (2024). However, some limitations remain. First, similarly to the original study, our RCT relied on a non‐clinical sample of women selected based on high weight and shape concerns. It requires additional research to test if the efficacy of the intervention also extends to individuals with an eating disorder diagnosis. We further acknowledge the limitations of the EDE‐Q global score in assessing behavioral eating disorder symptoms (e.g., bingeing and purging; exploratory analyses of the EDI‐2 bulimia subscale indicated however small to moderate effects of the intervention on bulimia scores at post‐assessment and follow‐up). Second, due to our study design, the relative effectiveness of the meaning‐centered intervention in relation to existing interventions for broader eating disorder symptoms has yet to be determined. Future research should investigate whether adding the current intervention to treatment‐as‐usual (TAU) increases effectiveness. Moreover, due to the relatively short follow‐up period of 4 weeks, the long‐term efficacy also remained unclear. Next, the dropout rate (around 30%) was higher than in the original study. Yet, this dropout rate was comparable to other individual meaning‐centered intervention studies (e.g., Breitbart et al. 2018) and rather underscores the low dropout rate in the original study. It is plausible that the social distancing context, which limited participants' daily activities and left more resources for psychological studies, contributed to fewer dropouts previously. Notably, in the current study, most dropouts did not occur during the intervention itself but resulted from non‐response to assessment invitations.

In conclusion, our study demonstrated replicational evidence for the efficacy of a meaning‐centered intervention in terms of higher life meaning for young women with weight and shape concerns in comparison to a waitlist control. In addition, participants in the intervention condition had lower internalizing and eating disorder symptoms immediately after the intervention and 4 weeks later, at least for those participants having relatively high eating disorder symptoms at baseline. Future research needs to investigate the utility of the intervention as an additional component in eating disorder treatments, while also assessing which specific intervention segments contributed to increases in life meaning.

## Conflicts of Interest

The authors declare no conflicts of interest.

## Supporting information

Supporting Information S1

## Data Availability

The data that support the findings of this study are available from the corresponding author upon reasonable request.
