# Supplementary material for: A Meaning‐Centered Intervention for Undergraduate Women With High Weight and Shape Concerns—Replication of a Randomized Controlled Trial
Source: Eur Eat Disord Rev. 2025 Jan 31;33(4):719–29. doi: 10.1002/erv.3175 (PMC12171670; doi:10.1002/erv.3175)
Supplement: Supplementary file 1 — Supporting Information S1 [file ERV-33-719-s001.docx]

**Supplementary Materials**

**Additional Information**

***Information About Dropouts***

Forty-nine participants started this research (i.e., filled in the baseline assessment and were allocated to a condition) but did not complete the post and/or follow-up assessment for various reasons that are reported in Figure 1 in the main manuscript (29.52%, intervention condition *n* = 25, waitlist condition *n* = 24). Condition did not affect the number of dropouts (χ^2^(1), *N* = 166, *p* > .05). The dropouts did not differ significantly in age and BMI from the completers, but had a lower WCS score (dropouts *M* = 53.37, *SD* = 13.29; completers *M* = 58.66, *SD* = 14.70; *t*[164] = 2.18, *p* < .05, *d* = .37). They further did not differ at baseline from those who completed the study on all outcome measures except the MEMS-Mattering, for which completers showed lower scores than dropouts (dropouts *M* = 3.41, *SD* = 1.26; completers *M* = 2.99, *SD* = 1.09, *t*[164] = -2.21, *p* < .05, *d* = -.38). The main analyses were done based on completers at the given assessment timepoint; including only those participants who completed all study parts did not result in different outcomes for the short-term effects analysis.

***Language Differences***

Since Kruskal-Wallis Tests indicated that participants who filled out the questionnaires in English scored significantly higher at T1 for the DASS total, the DASS subscales, and the EDE-Q than those who filled it in in Dutch, we included language as an independent variable and the interaction language*T1 into the according main analyses. Adding these predictors into the model did not change the outcomes for the analyses and they were left out subsequently.

***Covariate Effects***

Table 1 displays the effects of all covariate effects for the main analyses. All effect sizes can be considered large.

**Table 1**

*Covariate Effects for Primary and Secondary Analyses*

|  | ANCOVA | | | | | |
| --- | --- | --- | --- | --- | --- | --- |
|  | Post Assessment (T2) | | | Follow-up Assessment (T3) | | |
| T1 | F | *p* | *η_p_^2^* | F | *p* | *η_p_^2^* |
| MLQ-Presence | 130.80 | < .001 | .51 | 57.02 | < .001 | .33 |
| MEMS |  |  |  |  |  |  |
| Comprehension | 81.65 | < .001 | .39 | 66.08 | < .001 | .37 |
| Purpose | 142.26 | < .001 | .53 | 84.19 | < .001 | .43 |
| Mattering | 133.24 | < .001 | .51 | 110.39 | < .001 | .49 |
| EDE-Q | 92.30 | < .001 | .42 | 55.37 | < .001 | .33 |
| DASS-21 |  |  |  |  |  |  |
| Total | 95.58 | < .001 | .43 | 42.99 | < .001 | .27 |
| Depression | 95.29 | < .001 | .43 | 50.27 | < .001 | .31 |
| Anxiety | 109.86 | < .001 | .46 | 48.70 | < .001 | .30 |
| Stress | 34.62 | < .001 | .21 | 29.23 | < .001 | .20 |

*Note.* MLQ-Presence = Meaning in Life Questionnaire Presence subscale; MEMS = Multidimensional Existential Meaning Scale, subscales Comprehension, Purpose, Mattering; EDE-Q = Eating Disorder Examination-Questionnaire; DASS-21 = Depression Anxiety Stress Scales-21, Total score and subscales Depression, Anxiety, Stress.

**Additional Analyses**

***Intention-to-Treat Analyses***

Table 2 indicates the exact values for all intention-to-treat (ITT) analyses based on the ANCOVA models described in the main manuscript.

**Table 2**

*Exact ANCOVA ITT Outcomes.*

|  | T2 | | | T3 | | |
| --- | --- | --- | --- | --- | --- | --- |
|  | F | p | *η_p_^2^* | F | p | *η_p_^2^* |
| MLQ-Presence | 20.35 | < .001 | .11 | 6.31 | .007 | .04 |
| MEMS |  |  |  |  |  |  |
| Comprehension | 7.81 | .003 | .05 | 2.00 | .104 | .01 |
| Purpose | 9.95 | < .001 | .06 | 2.53 | .071 | .02 |
| Mattering | 8.10 | .003 | 0,.05 | .84 | .284 | .005 |
| EDE-Q | 17.20 | < .001 | .01 | 10.20 | < .001 | .06 |
| DASS-21 |  |  |  |  |  |  |
| Total | 23.43 | < .001 | .13 | 10.38 | < .001 | .06 |
| Depression | 14.11 | < .001 | .08 | 8.55 | .002 | .05 |
| Anxiety | 14.17 | < .001 | .08 | 6.46 | .006 | .04 |
| Stress | 12.61 | < .001 | .07 | 5.95 | .009 | .03 |

*Note.* MLQ-Presence = Meaning in Life Questionnaire Presence subscale; MEMS = Multidimensional Existential Meaning Scale, subscales Comprehension, Purpose, Mattering; EDE-Q = Eating Disorder Examination-Questionnaire; DASS-21 = Depression Anxiety Stress Scales-21, Total score and subscales Depression, Anxiety, Stress.

***Mixed Linear Modeling***

We further complemented our main analyses with a mixed linear modelling approach (mlm). Doing so allowed us to gain additional insight into time effects and provided a more complete picture of the effectiveness of our intervention. We used the package *lme4* in *R* version 4.4.1 (R Core Team, 2022) to conduct the mlm analyses, which takes correlations over time into account. This analysis deals with missing data based on Full Information Maximum Likelihood (FIML; e.g., Stroup, 2016), thus all 166 participants who were randomized into conditions were included in these analyses. Missingness completely at random (MCAR) was tested with Little's Missing Completely At Random test (Little, 1988) in the *naniar* package (*p* < .05), indicating that the data was not MCAR. Missingness at random was tested with a logistic regression (missingness for any variable as dependent variable with 0/1 and MLQ-P, MEMS-C, MEMS-M, MEMS-P, EDE-Q, DASS-D, DASS-A, DASS-S, and DASS-Total as predictors). Results indicated that the data was missing at random (all predictors *p* > .05). In case of assumption violations, we made use of robust standard errors in the *robustlmm* package. We included a random intercept for participants to account for individual differences in baseline scores. Time was coded as a categorical predictor to be able to assess interaction effects and compare between distinct timepoints, since we also did not expect a linear relationship between timepoints. Models with a random slope did not converge for any of the variables. Effect sizes (Cohen’s *d*) for differences between estimated marginal means (EMMs) were calculated with the eff_size function in the *emmeans* package and to account for the data being paired, an adjusted sigma was used (Lenth, 2024). Due to the exploratory nature of this analyses, we did not apply corrections for multiple testing.

**Meaning in Life Questionnaire – Presence Subscale.** A mlm was constructed to examine the effects of condition (waitlist vs. intervention) and time (baseline (T1), post-intervention (T2), follow-up (T3)) on the MLQ-P. Residual plots indicated no significant deviations from normality, and homoscedasticity was met across all levels of the predictors. Fixed effects can be found in Table 3. The variance of the random intercept for participants was estimated at σ² = 1.01, indicating individual differences in baseline MLQ-P scores. The model explained 10.5% of the variance in the outcome (marginal *R²* = .105), and 74.8% of the total variance (conditional *R²* = .748), indicating that the random intercept accounted for 64.8% of the variance in baseline MLQ-P scores. Residual plots indicated no significant deviations from normality, and homoscedasticity was met across all levels of the predictors. Post-hoc comparisons using Tukey’s HSD revealed that participants in the intervention condition showed significantly higher MLQ-P scores at T2 compared to the waitlist condition (*t*(263) = -4.92, Cohen’s *d* = 1.08, *p* < .001; intervention: *EMM* = 5.10 (*SE* = 0.14, 95% *CI* [4.83, 5.37]); waitlist: *EMM* = 4.14 (*SE* = 0.14, 95% *CI* [3.87, 4.41])), as well as at T3 (*t*(281) = -3.48, Cohen’s *d* = 0.79, *p* < .001; intervention: *EMM* = 4.85 (*SE* = 0.14, 95% *CI* [4.57, 5.12]); waitlist: *EMM* = 4.15 (*SE* = 0.14, 95% *CI* [3.87, 4.43])).

**Table 3**

***Fixed Effects for the MLQ-Presence Subscale***

|  | Estimate | Std. Error | *t*-value | *p*-value |
| --- | --- | --- | --- | --- |
| (Intercept) | 4.00 | 0.13 | 30.59 | < .001 |
| Condition (Intervention) | 0.18 | 0.18 | 0.95 | .34 |
| Time (T2) | 0.14 | 0.11 | 1.34 | .18 |
| Time (T3) | 0.15 | 0.11 | 1.34 | .18 |
| Condition*Time (T2) | 0.79 | 0.15 | 5.14 | < .001 |
| Condition*Time (T3) | 0.52 | 0.16 | 3.28 | < .01 |

**Multidimensional Existential Meaning Scale – Comprehension.** A mlm was constructed to examine the effects of condition (waitlist vs. intervention) and time (baseline (T1), post-intervention (T2), follow-up (T3)) on the MEMS-C. Residual plots indicated no significant deviations from normality, and homoscedasticity was met across all levels of the predictors. Fixed effects can be found in Table 4. The variance of the random intercept for participants was estimated at σ² = 0.64, indicating individual differences in baseline MEMS-C. The model explained 10.6% of the variance in the outcome and 72.1% of the total variance, indicating that the random intercept accounted for 61.5% of the variance in baseline MEMS-C scores. Post-hoc comparisons using Tukey’s HSD revealed that participants in the intervention condition showed significantly higher MEMS-C scores at T2 compared to the waitlist condition (*t*(273) = -4.16, Cohen’s *d* = 0.87, *p* < .001; intervention: *EMM* = 4.78 (*SE* = 0.11, 95% *CI* [4.56, 5.01]); waitlist: *EMM* = 4.11 (*SE* = 0.11, 95% *CI* [3.89, 4.34])), as well as at T3 (*t*(293) = -3.14, Cohen’s *d* = 0.68, *p* < .01; intervention: *EMM* = 4.56 (*SE* = 0.12, 95% *CI* [4.33, 4.79]); waitlist: *EMM* = 4.04 (*SE* = 0.12, 95% *CI* [3.81, 4.27])).

**Table 4**

***Fixed Effects for the MEMS-C***

|  | Estimate | Std. Error | *t*-value | *p*-value |
| --- | --- | --- | --- | --- |
| (Intercept) | 3.80 | 0.11 | 35.65 | < .001 |
| Condition (Intervention) | 0.24 | 0.15 | 1.63 | .10 |
| Time (T2) | 0.31 | 0.09 | 3.41 | < .001 |
| Time (T3) | 0.24 | 0.09 | 2.49 | < .05 |
| Condition*Time (T2) | 0.42 | 0.13 | 3.22 | < .01 |
| Condition*Time (T3) | 0.27 | 0.14 | 2.00 | < .05 |

**Multidimensional Existential Meaning Scale – Purpose.** A mlm was constructed to examine the effects of condition (waitlist vs. intervention) and time (baseline (T1), post-intervention (T2), follow-up (T3)) on the MEMS-P. Residual plots indicated no significant deviations from normality, and homoscedasticity was met across all levels of the predictors. Fixed effects can be found in Table 5. The variance of the random intercept for participants was estimated at σ² = 0.68, indicating individual differences in baseline MEMS-P. The model explained 5.0% of the variance in the outcome and 75.0% of the total variance, indicating that the random intercept accounted for 70% of the variance in baseline MEMS-P scores. Post-hoc comparisons using Tukey’s HSD revealed that participants in the intervention condition showed significantly higher MEMS-P scores at T2 compared to the waitlist condition (*t*(256) = -3.37, Cohen’s *d* = 0.74, *p* < .001; intervention: *EMM* = 5.41 (*SE* = 0.11, 95% *CI* [5.19, 5.64]); waitlist: *EMM* = 4.88 (*SE* = 0.11, 95% *CI* [4.67, 5.10])), as well as at T3 (*t*(274) = -2.28, Cohen’s *d* = 0.53, *p* < .05; intervention: *EMM* = 5.28 (*SE* = 0.11, 95% *CI* [5.05, 5.50]); waitlist: *EMM* = 4.91 (*SE* = 0.11, 95% *CI* [4.68, 5.13])). Note that the fixed effect of Condition*Time (T3) is non-significant in Table 4; indicating that the difference in MEMS-P between conditions at T3 is not statistically different from the reference category (T1) in the overall model.

**Table 5**

***Fixed Effects for the MEMS-P***

|  | Estimate | Std. Error | *t*-value | *p*-value |
| --- | --- | --- | --- | --- |
| (Intercept) | 4.80 | 0.11 | 45.35 | < .001 |
| Condition (Intervention) | 0.17 | 0.15 | 1.16 | .25 |
| Time (T2) | 0.09 | 0.08 | 1.07 | .29 |
| Time (T3) | 0.11 | 0.09 | 1.27 | .20 |
| Condition*Time (T2) | 0.36 | 0.12 | 2.99 | < .01 |
| Condition*Time (T3) | 0.20 | 0.12 | 1.57 | .12 |

**Multidimensional Existential Meaning Scale – Mattering.** A mlm was constructed to examine the effects of condition (waitlist vs. intervention) and time (baseline (T1), post-intervention (T2), follow-up (T3)) on the MEMS-M. Residual plots indicated no significant deviations from normality, and homoscedasticity was met across all levels of the predictors. Fixed effects can be found in Table 6. The variance of the random intercept for participants was estimated at σ² = 1.05, indicating individual differences in baseline MEMS-M. The model explained 7.2% of the variance in the outcome and 76.9% of the total variance, indicating that the random intercept accounted for 69.7% of the variance in baseline MEMS-M scores. Post-hoc comparisons using Tukey’s HSD revealed that participants in the intervention condition showed significantly higher MEMS-M scores at T2 compared to the waitlist condition (*t*(251) = -4.21, Cohen’s *d* = 0.98, *p* < .001; intervention: *EMM* = 3.91 (*SE* = 0.14, 95% *CI* [3.64, 4.19]); waitlist: *EMM* = 3.10 (*SE* = 0.14, 95% *CI* [2.83, 3.36])), as well as at T3 (*t*(2269) = -2.51, Cohen’s *d* = 0.60, *p* < .05; intervention: *EMM* = 3.71 (*SE* = 0.14, 95% *CI* [3.43, 3.98]); waitlist: *EMM* = 3.21 (*SE* = 0.14, 95% *CI* [2.93, 3.49])). Note that the fixed effect of Condition*Time (T3) is non-significant in Table 5; indicating that the difference in MEMS-M between conditions at T3 is not statistically different from the reference category (T1) in the overall model.

**Table 6**

***Fixed Effects for the MEMS-M***

|  | Estimate | Std. Error | *t*-value | *p*-value |
| --- | --- | --- | --- | --- |
| (Intercept) | 2.98 | 0.13 | 22.82 | < .001 |
| Condition (Intervention) | 0.26 | 0.18 | 1.40 | .16 |
| Time (T2) | 0.11 | 0.10 | 1.13 | .26 |
| Time (T3) | 0.23 | 0.11 | 2.14 | < .05 |
| Condition*Time (T2) | 0.56 | 0.14 | 3.89 | < .001 |
| Condition*Time (T3) | 0.24 | 0.15 | 1.61 | .11 |

**Eating Disorder Examination-Questionnaire.** A mlm was constructed to examine the effects of condition (waitlist vs. intervention) and time (baseline (T1), post-intervention (T2), follow-up (T3)) on the EDE-Q. Residual plots indicated no significant deviations from normality, and homoscedasticity was met across all levels of the predictors. Fixed effects can be found in Table 7. The variance of the random intercept for participants was estimated at σ² = 0.86, indicating individual differences in baseline EDE-Q scores. The model explained 12.5% of the variance in the outcome and 72.9% of the total variance, indicating that the random intercept accounted for 60.4% of the variance in baseline EDE-Q scores. Post-hoc comparisons using Tukey’s HSD revealed that participants in the intervention condition showed significantly lower EDE-Q scores at T2 compared to the waitlist condition (*t*(272) = 4.78, Cohen’s *d* = 1.01, *p* < .001; intervention: *EMM* = 1.96 (*SE* = 0.13, 95% *CI* [1.70, 2.22]); waitlist: *EMM* = 2.84 (*SE* = 0.13, 95% *CI* [2.59, 3.10])), as well as at T3 (*t*(292) = 4.11, Cohen’s *d* = 0.89, *p* < .001; intervention: *EMM* = 2.02 (*SE* = 0.13, 95% *CI* [1.76, 2.29]); waitlist: *EMM* = 2.81 (*SE* = 0.14, 95% *CI* [2.54, 3.07])).

**Table 7**

***Fixed Effects for the EDE-Q***

|  | Estimate | Std. Error | *t*-value | *p*-value |
| --- | --- | --- | --- | --- |
| (Intercept) | 2.95 | 0.12 | 23.91 | < .001 |
| Condition (Intervention) | 0.02 | 0.17 | 0.14 | .89 |
| Time (T2) | -0.11 | 0.11 | -1.04 | .30 |
| Time (T3) | -0.14 | 0.11 | -1.30 | .20 |
| Condition*Time (T2) | -0.91 | 0.15 | -6.02 | < .001 |
| Condition*Time (T3) | -0.81 | 0.16 | -5.13 | < .001 |

**Depression Anxiety Stress – Scales – Total.** A mlm was constructed to examine the effects of condition (waitlist vs. intervention) and time (baseline (T1), post-intervention (T2), follow-up (T3)) on the DASS-Total. Residual plots and homoscedasticity tests indicated mild significant deviations from normality, which is why we chose the robust standard error method. Fixed effects can be found in Table 8. The variance of the random intercept for participants was estimated at σ² = 103.74, indicating individual differences in baseline DASS-Total scores. The model explained 5.8% of the variance in the outcome and 73.4% of the total variance, indicating that the random intercept accounted for 67.6% of the variance in baseline DASS-Total scores. Post-hoc comparisons using Tukey’s HSD revealed that participants in the intervention condition showed significantly lower DASS-Total scores at T2 compared to the waitlist condition (*SE* = 2.04, *z* = 3.19, Cohen’s *d* = 0.66, *p* < .01; intervention: *EMM* = 17.4 (*SE* = 1.45, 95% *CI* [14.6, 20.2]); waitlist: *EMM* = 23.9 (*SE* = 1.43, 95% *CI* [21.1, 26.7])), as well as at T3 (*SE* = 2.09, *z* = 2.44, Cohen’s *d* = 0.52, *p* < .05; intervention: *EMM* = 18.9 (*SE* = 1.47, 95% *CI* [16.0, 21.7]); waitlist: *EMM* = 23.9 (*SE* = 1.48, 95% *CI* [21.0, 26.8])).

**Table 8**

***Fixed Effects for the DASS-Total***

|  | Estimate | Std. Error | *t*-value | *p*-value |
| --- | --- | --- | --- | --- |
| (Intercept) | 24.37 | 1.36 | 17.89 | < .001 |
| Condition (Intervention) | 0.98 | 1.91 | 0.51 | .61 |
| Time (T2) | -0.49 | 1.11 | -0.44 | .66 |
| Time (T3) | -0.43 | 1.18 | -0.36 | .72 |
| Condition*Time (T2) | -7.47 | 1.60 | -4.68 | < .001 |
| Condition*Time (T3) | -6.07 | 1.66 | -3.65 | < .001 |

**Depression Anxiety Stress – Scales – Depression.** A mlm was constructed to examine the effects of condition (waitlist vs. intervention) and time (baseline (T1), post-intervention (T2), follow-up (T3)) on the DASS-D. Residual plots and homoscedasticity tests indicated mild deviations from normality, which is why we chose the robust standard error method. Fixed effects can be found in Table 9. The variance of the random intercept for participants was estimated at σ² = 16.52, indicating individual differences in baseline DASS-D scores. The model explained 3.9% of the variance in the outcome and 68.6% of the total variance, indicating that the random intercept accounted for 64.7% of the variance in baseline DASS-D scores. Post-hoc comparisons using Tukey’s HSD revealed that participants in the intervention condition showed significantly lower DASS-D scores at T2 compared to the waitlist condition (*SE* = .84, *z* = 2.88, Cohen’s *d* = 0.57^[[1]](#footnote-1)^, *p* < .01; intervention: *EMM* = 4.90 (*SE* = 0.60, 95% *CI* [3.72, 6.08]); waitlist: *EMM* = 7.33 (*SE* = 0.59, 95% *CI* [6.17, 8.49])), as well as at T3 (*SE* = .87, *z* = 2.60, Cohen’s *d* = 0.53, *p* < .01; intervention: *EMM* = 5.70 (*SE* = 0.61, 95% *CI* [4.49, 6.90]); waitlist: *EMM* = 7.96 (*SE* = 0.61, 95% *CI* [6.75, 9.17])).

**Table 9**

***Fixed Effects for the DASS-D***

|  | Estimate | Std. Error | *t*-value | *p*-value |
| --- | --- | --- | --- | --- |
| (Intercept) | 7.27 | 0.56 | 12.97 | < .001 |
| Condition (Intervention) | -0.38 | 0.79 | -0.48 | .64 |
| Time (T2) | 0.06 | 0.49 | 0.11 | .91 |
| Time (T3) | 0.69 | 0.52 | 1.32 | .19 |
| Condition*Time (T2) | -2.06 | 0.71 | -2.92 | < .01 |
| Condition*Time (T3) | -1.89 | 0.74 | -2.57 | < .05 |

**Depression Anxiety Stress – Scales – Anxiety.** A mlm was constructed to examine the effects of condition (waitlist vs. intervention) and time (baseline (T1), post-intervention (T2), follow-up (T3)) on the DASS-A. Residual plots and homoscedasticity tests indicated mild deviations from normality, which is why we chose the robust standard error method. Fixed effects can be found in Table 10. The variance of the random intercept for participants was estimated at σ² = 14.41, indicating individual differences in baseline DASS-D scores. The model explained 4.3% of the variance in the outcome and 70.7% of the total variance, indicating that the random intercept accounted for 66.4% of the variance in baseline DASS-D scores. Post-hoc comparisons using Tukey’s HSD revealed that participants in the intervention condition showed significantly lower DASS-A scores at T2 compared to the waitlist condition (*SE* = .77, *z* = 2.44, Cohen’s *d* = 0.50, *p* < .05; intervention: *EMM* = 5.43 (*SE* = 0.55, 95% *CI* [4.35, 6.51]); waitlist: *EMM* = 7.31 (*SE* = 0.54, 95% *CI* [6.25, 8.38])), but not at T3 (*SE* = .80, *z* = 1.57, *p* > .05).

**Table 10**

***Fixed Effects for the DASS-A***

|  | Estimate | Std. Error | *t*-value | *p*-value |
| --- | --- | --- | --- | --- |
| (Intercept) | 7.27 | 0.52 | 14.08 | < .001 |
| Condition (Intervention) | 0.67 | 0.72 | 0.92 | .36 |
| Time (T2) | .05 | 0.44 | 0.11 | .91 |
| Time (T3) | -0.67 | 0.46 | -1.44 | .15 |
| Condition*Time (T2) | -2.56 | 0.63 | -4.07 | < .001 |
| Condition*Time (T3) | -1.92 | 0.66 | -2.92 | < .01 |

**Depression Anxiety Stress – Scales – Stress.** A mlm was constructed to examine the effects of condition (waitlist vs. intervention) and time (baseline (T1), post-intervention (T2), follow-up (T3)) on the DASS-S. Residual plots indicated no significant deviations from normality, but homoscedasticity tests indicated mild deviations from normality, which is why we chose the robust standard error method. Fixed effects can be found in Table 11. The variance of the random intercept for participants was estimated at σ² = 12.15, indicating individual differences in baseline DASS-S scores. The model explained 6.2% of the variance in the outcome and 60.8% of the total variance, indicating that the random intercept accounted for 54.6% of the variance in baseline DASS-S scores. Post-hoc comparisons using Tukey’s HSD revealed that participants in the intervention condition showed significantly lower DASS-S scores at T2 compared to the waitlist condition (*SE* = .79, *z* = 2.64, Cohen’s *d* = 0.49, *p* < .01; intervention: *EMM* = 6.92 (*SE* = 0.57, 95% *CI* [5.82, 8.03]); waitlist: *EMM* = 9.01 (*SE* = 0.55, 95% *CI* [7.93, 10.09])), but not at T3 (*SE* = .82, *z* = 1.83, *p* > .05).

**Table 11**

***Fixed Effects for the DASS-S***

|  | Estimate | Std. Error | *t*-value | *p*-value |
| --- | --- | --- | --- | --- |
| (Intercept) | 9.54 | 0.52 | 18.43 | < .001 |
| Condition (Intervention) | 0.89 | 0.73 | 1.22 | .22 |
| Time (T2) | -0.53 | 0.51 | -1.04 | .30 |
| Time (T3) | -0.37 | 0.54 | -0.68 | .50 |
| Condition*Time (T2) | -2.97 | 0.73 | -4.06 | < .001 |
| Condition*Time (T3) | -2.38 | 0.76 | -3.12 | < .01 |

**References**

Lenth, R. V. (2024). *emmeans: Estimated Marginal Means, aka Least-Squares Means*. R package version 1.10.4.900001, https://rvlenth.github.io/emmeans/.

Little, R. J. A. (1988). A test of Missing Completely at Random for multivariate data with missing values. *Journal of the American Statistical Association*, *83*(404), 1198–1202. https://doi.org/10.1080/01621459.1988.10478722.

R Core Team. (2022). *R: A language and environment for statistical computing.* In R Foundation for Statistical Computing. https://www.R-project.org/

Stroup, W. W. (2016). *Generalized linear mixed models: Modern concepts, methods and applications*. CRC Press.

1. Cohen’s d was based on the non-robust model, since the robust model does not indicate df, making it impossible to calculate Cohen’s d. This issue occurred for all robust models (i.e., DASS-D, DASS-A, DASS-S, and DASS-Total). [↑](#footnote-ref-1)
